# Exploratory Factor Analysis of the Adversity Appraisal Questionnaire (AAQ)

**DOI:** 10.1007/s10862-025-10219-7

**Published:** 2025-05-26

**Authors:** Jane Jiyoun Lee, Gabrielle Lowenthal, Yo Jackson

**Affiliations:** 1 Child Maltreatment Solutions Network, Social Science Research Institute, Pennsylvania State University, 202 Henderson Building, University Park, PA 16802 USA; 2 Department of Psychology, The Pennsylvania State University, 140 Moore Building, University Park, PA 16802 USA

**Keywords:** Adversity, Appraisal, Factor analysis, Trauma, Hostile attribution bias

## Abstract

Appraisal refers to a cognitive process through which individuals perceive and interpret a given event. This study evaluated the factorial structure of the 22-item Adversity Appraisal Questionnaire (AAQ) developed to assess the appraisal styles of an adversity exposed, community sample in response to their experiences with potentially stressful life events. Given that current assessment tools for appraisal are limited by scope and range of appraisal elements, the AAQ was developed as an amalgam of all currently available appraisal measures and includes questions about how adults think and feel about potentially adverse events in their lives. The measure also asks why a given event was or was not impactful or important for the respondent. Exploratory factor analyses (EFA) reliably identified a three-factor solution indicating three broad dimensions of appraisal: Emotional Distress, Perceived Controllability, and Perceived Threat. Internal consistency estimates for subscales were evaluated using Cronbach’s α (Emotional Distress: α = 0.94, Perceived Controllability: α = 0.79, Perceived Threat: α = 0.75). Findings suggested that the AAQ is a psychometrically reliable instrument for assessing adults’ appraisal styles and may be useful in studies requiring a comprehensive measurement of adversity appraisal. To document how the new three appraisal factors might be related to other important mental health cognitive processes, the study also examined the relation to hostile attribution biases as an example. The findings indicated that emotional distress appraisals are associated with hostile attribution biases. Recommendations for future research are provided.

## Introduction

Reactions to potentially adverse experiences are not always predictable. Two people exposed to the same event can and do have different responses. Differences in how each person appraises the event is one possible mechanism that may explain this response variability. Appraisal is defined as a cognitive process by which individuals perceive, interpret, and evaluate a stressor (Lazarus & Folkman, 1984). Appraisal theory posits that subjective interpretations of events are what lead to behavioral and emotional reactions to events (Lazarus & Folkman, 1984). Empirical studies have found appraisal to be significantly associated with anxiety (Gillanders et al., 2015), posttraumatic stress disorder (PTSD) (Kichline et al., 2017), and depression (Gunthert et al., 2007), sometimes even after controlling for the adverse event (e.g., DePrince et al., 2011). This suggests that it may not be the exposure itself but the interpretation of the event that is the most predictive of how one will respond, highlighting the necessity to understand subjective interpretations of stressful events to fully understand the consequences that follow afterwards. However, research on exposure to adversity and subsequent outcomes very seldomly focuses on the individual’s interpretation of the experiences, perhaps limiting understanding of key factors that contribute to differences in response to events.

### Limitations of Existing Appraisal Assessment Tools

Pre-existing appraisal measurement tools have been found to be lacking in scope to comprehensively capture the complexity of how one interprets adverse experiences (Gusler et al., 2023). Moreover, the available measures tend to have a great deal of specificity in terms of the types of adversity that the subjects are appraising (Gusler et al., 2021). To illustrate, different measures exist for accidental injury (Hepp et al., 2005), infertility treatment (Knoll et al., 2009), and chronic pain (Janowski et al., 2010), among others, emphasizing the circumstantial specificity of appraisal measurement tools. In addition to the variation in events that each tool is designed to appraise, there is also wide variability in the theory-based categories and dimensions (i.e., cognitive, emotional, primary, secondary, etc.) that make up the appraisal construct as well. For example, existing appraisal tools show inconsistency in the number and dimensions of appraisal that are measured. For example, the Coping Strategies Questionnaire (CSQ; Monticone et al., 2014) which measures appraisal of chronic pain, includes appraisal dimensions of Distraction, Praying, and Catastrophizing, among others. On the other hand, the Posttraumatic Cognitions Inventory (PTCI; Foa et al., 1999), developed to appraise accidental injury, includes dimensions of Negative Cognitions About Self, Negative Cognitions About the World, and Self-Blame.

However, research has indicated that there is a degree of similarity in the way individuals perceive stressful situations, in that they possess an inherent characteristic that predisposes them to consistently view stressors as either challenging or threatening Kilby et al. (2018). In this approach, individuals have shown to possess dispositional appraisal styles that they use for a range of different stressors (Lazarus & Folkman, 1984). For example, one study found that women with resilient appraisal styles that involved seeking for support and avoiding isolation applied this appraisal style to the loss of their spouse, experiencing better life adjustment and satisfaction after one month of their spouse’s death compared to those who did not have this resilient appraisal style (Rossi et al., 2005). This indicates that it may not be necessary for researchers to develop measurement tools for each type of adversity relevant to their research questions. Rather, the development of a consistent tool that can reliably assess for appraisal patterns across multiple events, and comprehensively capture a myriad of appraisal dimensions based on theory, is needed.

Despite the dearth of measurement tools that are suitable for use for varied adverse event types, the Stress Appraisal Measure (SAM; Peacock & Wong, 1990) and the Trauma Appraisal Questionnaire (TAQ; DePrince et al., 2010) assesses potential stressors in varied settings. To illustrate, the SAM was created to assess appraisal of stressors that are relatively more common, such as searching for a new job or taking an exam (Peacock & Wong, 1990). In contrast, the TAQ was specifically developed to evaluate appraisals of potentially life-threatening traumatic events like physical or sexual abuse (DePrince et al., 2010). Consequently, the TAQ and SAM have been designed to appraise different types of stress and cannot be used for the broad spectrum of both common stressors and more serious traumatic events.

### Theoretical Perspectives of Measuring Appraisal

Appraisal can be categorized into two main types- primary and secondary appraisal (Lazarus & Folkman, 1984). Primary appraisal is the way individuals interpret the significance of the event to their well-being (e.g., irrelevant, benign-positive, or stressful). Secondary appraisal is how individuals react to the adverse event (e.g., emotion-focused coping or blame) and their perceptions of how effectively they are able to respond to the event (e.g., control and self-efficacy). Some researchers have extended beyond primary and secondary categories to classify appraisals into smaller dimensions of emotional responses (e.g., loss, anger, threat). Different types of appraisal processes are then associated with specific coping responses, which involves either modifying the problem by generating steps to solve the issue or managing the emotional distress that is associated with the situation (Lazarus & Folkman, 1984). So far, no measure is comprehensive enough to capture both one’s interpretation and response to events (Gusler et al., 2021).

### Adversity Appraisal and Hostile Attribution Bias (HAB)

One construct that is especially relevant to appraisal is hostile attribution biases (HAB). Exposure to adverse events is related to maladaptive thoughts such as overgeneralizations (perceiving one negative event as a never-ending pattern of defeat; Ehlers & Clark, 2000), catastrophizing (exaggerating the significance of one event; Muran & Motta, 1993), and selective abstraction (paying attention to one or part of the details and failing to see the whole picture; Nibaruta et al., 2022). Such cognitive biases may also result in variations in how individuals process information from the environment and interpret social cues. In particular, exposure to adversity has been found to lead to experience-specific alterations in information processing (Griffith et al., 2021). Specifically, HAB, defined as the tendency to perceive the intentions of others as hostile despite no external cues that suggest such intentions, may emerge when negative experiences and cognitive schemas that represent other people and events trigger associations with previous experiences (Guerra & Huesmann, 2004). Taken together, appraisal styles may also be related to broader interpretation of typical daily social cues, whereby cognitive biases and HAB are associated.

According to social-cognitive theorists, individuals with elevated levels of HAB face challenges with social interactions, in turn reinforcing their HAB. This is because individuals who respond with negative biases are more likely to encounter difficult social situations that reinforce their negative beliefs about others’ intentions, making it difficult for them to develop more positive strategies for interacting with others (Verhoef et al., 2019). In this light, individuals with negative appraisal styles may also have trouble accurately interpretating social cues. These studies suggest that if adversity appraisals and HAB are associated, individuals’ appraisal styles may have implications for not only the way important traumatic events are interpreted, but how daily social interactions take place as well.

### The Present Study

Both appraisal theory (Lazarus & Folkman, 1984) and previous empirical evidence (e.g., DePrince et al., 2010) support the importance of incorporating appraisal into research which aims to understand processes connecting adverse experiences and developmental health outcomes. For example, negative appraisal has been associated with greater risk for physical (O’Loughlin et al., 2019) and mental health problems (Khoury et al., 2021). Stress threatens one’s well-being when an individual perceives their environment to exceed their capacity to manage it (Lazarus & Folkman, 1984). The cognitive appraisal of the stressful event, then, is what determines personal meaning and the level of affliction one experiences (Holroyd & Lazarus, 1982). This is why a situation may be interpreted as ‘stressful’ for one person but not for another.

In summary, the need for a new, standardized appraisal measure arises from several critical gaps identified in the current research. First, existing studies overwhelmingly rely on self-created measures (e.g., Herrero et al., 2008; Kennedy et al., 2012) designed for specific research questions, resulting in significant variability in how appraisals of adversity are assessed. This inconsistency makes it challenging to draw generalized conclusions or compare findings across studies. One scoping review (Gusler et al., 2021*) assessed 88 articles from three research databases, comparing adults’ appraisals of adversity. This scoping review indicates that over 54 distinct tools for appraising stressful events have been developed, further highlighting the need for a measurement tool that can assess appraisal styles across a range of events rather than focusing on one very specific type of event. Moreover, the lack of standardized appraisal tools contributes to difficulties in ensuring the generalizability and reproducibility of findings. Without a reliable, multidimensional measure of cognitive appraisal that accounts for both primary (e.g., threat, harm) and secondary (e.g., coping ability, resources) appraisals, researchers are unable to consistently capture the full spectrum of reactions to adversity.

Second, many appraisal measures are often brief (comprising only 1–2 items), which likely fails to capture the complex, multidimensional nature of how individuals process adversity. This limitation, coupled with the fact that appraisal measures tend to focus on a narrow range of dimensions (e.g., threat, stressfulness, and self-blame), overlooks the nuanced ways in which individuals appraise traumatic or adverse experiences.

Lastly, issues of diversity and equity arise, as many studies fail to report participant demographic information such as race, ethnicity, or income, which are essential for understanding how adversity may be appraised differently across diverse populations. A comprehensive, validated appraisal tool that measures reactions to adversity exposure across a diverse range of event types would address these limitations by offering a consistent framework for assessing reactions to adversity, facilitating comparisons across studies, and promoting inclusivity in the research process.

In light of such need, the Adversity Appraisal Questionnaire (AAQ) is an assessment tool newly constructed for individuals to appraise not one specific type of event, but across a myriad of different event types, allowing versatility to accommodate a wide range of adverse events. This study evaluated the factorial structure of the AAQ. Based on the original items of the AAQ, it was expected that some dimensions of appraisal such as Threat, Uncontrollability, Emotional Distress, and Self-Blame that are present in existing measures of appraisal may also arise as factors in the AAQ.

## Methods

### Participants

Participants included 105 caregivers (*M*_*age*_ = 31.41, *SD* = 8.29) of children between the ages of three- and five-years old residing in a large Midwestern city. All participants were recruited from the Preschoolers’ Adjustment and Intergenerational Risk (PAIR) project. PAIR is a federally-funded, five-year longitudinal study that aims to understand how adversity exposure in children and caregivers impacts a child’s development. Participants were recruited from the local community through partnerships with Head Start programs, the Department of Social Services, and agencies serving low-income families. Approximately 50% of the participants reported an annual income level below $10,000, and approximately 80% of participants reported an annual income level below $30,000. Regarding participants’ race/ethnicity, the majority of participants identified as Black or African American (79.6%), followed by Caucasian (12.4%), Multiracial (3.5%), American Indian/Alaska Native (2.7%), or Other (1.8%). Approximately 32% graduated from some high school/GED, followed by some college (25.8%), and then trade school or community college (16.7%) as their highest level of education. Over half of the sample were single (68.3%). The majority of the sample were female (91.4%). Further details about the analytic sample are presented in below in Table 1.

**Table 1:** Sample demographic characteristics

Variable	Range	*N*	Mean (SD)	Percent (%)
**Age**	21–70		31.41 *(8.29)*	
**Number of Traumatic Events Exposed to during adulthood**	0–34		9.00 *(7.00)*	
**Hostile Attribution Biases**				
General Hostile Attributions	0–27		12.50 *(6.33)*	
General Hostile Responding	0–24		9.37 *(6.15)*	
Child Specific Hostile Attribution	0–30		11.53 *(7.26)*	
**Gender**				
Female		96		91.4
Male		6		5.7
**Total Family Income**				
$10,000 or less		65		52.4
$10,001-$20,000		22		17.7
$20,001-$30,000		24		19.4
$30,001-$40,000		4		3.2
$40,001-$50,000		3		2.4
$50,001 or more		6		4.8
**Race/ethnicity**				
American Indian or Alaska Native		3		2.7
Black or African American		90		79.6
White or Caucasian		14		12.4
Multiracial		4		3.5
Other		2		1.8
**Highest level of education**				
Some Grade School		5		4.2
Some High School		20		16.7
High School Graduate or GED		38		31.7
Trade School or Community College Graduate		20		16.7
Some College		31		25.8
Four-Year Degree College Graduate		4		3.3
Graduate or Professional School		2		1.7
**Marital Status**				
Married		15		12.2
Divorced/Separated		9		7.3
Widowed		1		0.8
Remarried		2		1.6
Single		84		68.3
Other		12		9.8

### Procedures

Data collection sessions occurred at community venues conveniently located near families’ residences. The data collection sessions varied in duration from three to five hours, during which children engaged in research activities for around one hour, and supervised free play for the remaining hours. To accommodate the extended duration of data collection, participants were provided with a range of snacks and water, childcare services for participating children and their siblings, and were encouraged to take frequent breaks. A pilot phase of data collection involved approximately 40 families who successfully completed the study assessments and laboratory tasks, ensuring that the data collection process was feasible and did not overly burden the participants. All data collection procedures and methods were approved by the Institutional Review Board of the investigator’s university. After completing the informed consent process, parents in the project completed the study using paper and pencil to answer questions, with questions being read aloud if they showed difficulties with reading or comprehension. After completing the study measure and activities, participants were debriefed, and gift cards were used to compensate for their time and participation.

### Measures

*Demographics.* Participants provided self-reported demographic information including their gender, age, annual income, race/ethnicity, education level, and marital status.

*Adversity Appraisal Questionnaire (AAQ).* Appraisal of adversity was measured using the Adversity Appraisal Questionnaire (AAQ), a tool constructed to capture participants’ patterns of appraisal across adverse events. Participants administered the AAQ were asked to list all the important events in their life, of which they were asked to choose one event to answer the follow-up questions of the questionnaire. In the larger project, participants were assessed for exposure to 45 different potentially adverse life events. These adverse events include both potentially less severe adversities such as moving to a new country, as well as more threatening events, such as physical abuse. The development of the AAQ involved scoping existing measurement tools such as the Trauma Appraisal Questionnaire (TAQ; DePrince et al., 2010) and the Stress Appraisal Measure (SAM; Peacock & Wong, 1990), which have demonstrated strong psychometric reliability and construct validity. Lastly, a comprehensive list of appraisal dimensions (e.g., importance, expectedness, agency, and familiarity; Frijda, 1987) were utilized in the development of the AAQ. As a result, each item in the AAQ was designed to assess a unique and potentially relevant appraisal dimension. In order to ensure the questionnaire content was appropriate and relevant to measure adults’ trauma appraisals, 7 experts in trauma, social welfare, and psychology were identified by the research team and invited via email to participate in a content validity survey. Six experts held positions in academia (including 3 postdoctoral fellows, 2 full professors, and 1 graduate research student). Considering that the questionnaire is an appraisal tool for exposure to trauma and adversity, one field expert was additionally included as well (clinician practicing in clinical rehabilitation and mental health counseling). Experts were asked to: (1) Rate the clarity (i.e., items are clear and concise, reducing variability in responses), validity (items represent the domain of the construct being measured), and relevance of the AAQ items in representing the proposed construct on a 4-point content validity scale; (2) Suggest item revisions (including removal of redundant items); (3) Suggest domain name and domain definition revisions; and/or (4) Suggest additional items for the AAQ. Based on the content validity feedback from experts and discussion with the research team, additional changes were made. First, suggestions related to the clarity of the items were appropriately edited so that the wording was concise and less ambiguous, reducing variability in responses (e.g., “The event is important for me because it had a positive impact on my life” was changed to “The event is important for me because it had a positive impact on my life despite it being a difficult experience”). Second, items that were identified as redundant were suggested to be removed from the questionnaire, so that individuals answering the questionnaire would not experience fatigue from the length of the questionnaire (e.g., “Thinking about the event makes me feel: Happy/Joyful” was removed for being too redundant with “Thinking about the event makes me feel: Sadness/grief” if it were to be reverse coded.) Lastly, experts recommended a space for text entry be added for participants to describe in greater detail their emotions when thinking about a particular event. As such, a text entry question was added to the end of the questionnaire, which read “Thinking about this event, what other emotions do you feel, that were not previously listed?” Due to the qualitative nature of this question, it was not a part of the EFA analyses.

The AAQ is a 22-items scale answered on a 5-point scale from 1 “Strongly Disagree” to 5 “Strongly Agree.” Each of the 22 items asked participants to appraise how they feel when thinking about the event (e.g., “Thinking about the event makes me feel embarrassed”) or why the event is impactful or important for them (e.g., “The event is important for me because: It affected someone else’s well-being”). A full list of items of the AAQ is presented below in Table 2.

**Table 2:** Items of the adversity appraisal questionnaire (AAQ)

Question Number	Question
1	The event is important for me because it affected someone else’s well-being
2	The event is important for me because it had a positive impact on my life despite it being a difficult experience
3	The event is important for me because it had a negative impact on my life
4	The event is important for me because it had a lasting impact for me
5	The event is important for me because I felt that my life was threatened because of the event
6	The event is important for me because it happened to me because of the sort of person I am
7	The event is important for me because I couldn’t stop this event from happening to me
8	The event is important for me because I was responsible for what happened
9	I understand what happened and what the consequences were
10	When it happened, I could predict how the event was going to end
11	The event was expected. Meaning, I knew the event was going to happen
12	Things I expected to happen during the event did actually happen.
13	Thinking about the event makes me feel lonely
14	Thinking about the event makes me feel angry
15	Thinking about the event makes me feel happy/joyful
16	Thinking about the event makes me feel embarrassed
17	Thinking about the event makes me feel anxious/afraid
18	Thinking about the event makes me feel sadness/grief
19	Thinking about the event makes me feel guilty
20	Thinking about the event makes me feel shameful
21	Thinking about the event makes me feel pride
22	Thinking about the event makes me feel confused

*Adult Trauma Questionnaire*. As this is a high adversity exposed sample, the Adult Trauma Questionnaire has been used to collect information on the level of stressful life events the sample has been exposed to. The Adult Trauma Questionnaire has been constructed by the PAIR project and covers 45 different potentially traumatic life events from previous measures with established validity and reliability (e.g., the ACEs questionnaire, Life Events Checklist, etc.). The Adult Trauma Questionnaire spanned a broad and comprehensive range of event types such as witnessing and experiencing violence, unemployment, and unwanted sexual contact or harassment. A summary of the included sample’s adulthood trauma can be found in the appendix. On average, participants endorsed to have experienced 9 events, with some participants endorsing as many as 34 events. As the larger project targets a community sample with relatively high levels of stress, participants had many potential events they were able to appraise.

*Hostile Attribution Bias (HAB).* The Parental Hostile Attribution Questionnaire (Parental HAQ; Halligan et al., 2007) has been administered to participants in order to measure their general hostile tendencies, child-specific hostile tendencies, and aggressive responding. The Parental HAQ presents six general ambiguous social scenarios which involves interactions with other individuals (e.g., someone bumping into them or another driver on the road accelerating to drive in front of them). After each vignette, participants are asked a series of follow up questions that represent potential aggressive responses (e.g., bumping into the person that bumped into them or making a rude gesture to the other driver) or hostile interpretations (e.g., thinking that the other individual deliberately did the ambiguous action), which are rated on a six-point Likert scale from (*0 = Extremely Unlikely* to *5 = Extremely Likely)* of how likely they are to respond aggressively and how likely they are to interpret the situation as hostile in intent. Following the six general ambiguous scenarios, participants then responded to six child-specific ambiguous situations (e.g., their child not responding when they tell them goodnight or breaking a toy). For each child-specific vignette, the participants again indicated their interpretation (e.g., thinking that their child deliberately performed the ambiguous action to purposely make them mad) on a six-point Likert scale from (*0 = Extremely Unlikely to 5 = Extremely Likely).* Halligan and colleagues (2007) have conducted a small-scale validation study of the HAQ, and examined concurrent validity via relations with the Spielberger State-Trait Anger Expression Inventory, Version 2 (STAXI-2), a measure of angry feelings and their expression with established reliability and validity (Spielberger, 2002). Hostile attribution bias scores correlated with Trait Anger scores on the STAXI-2 (*r =*.74, *p* <.001) and hostile attribution responding correlated with the Anger-Expression scale (*r =*.51, *p <*.05). For the present study, the internal consistently for each subscale as measured by Cronbach’s α ranged from acceptable to good (e.g., General Hostile Interpretation α = 0.78, Child-Specific Hostile Interpretation α = 0.78, and Aggressive Respondingα = 0.83). 

### Data Analysis

First, frequency and descriptive statistics summarizing the characteristics of the sample and study variables were completed. A summary of participants’ gender, age, income level, race/ethnicity, education level, and marital status are provided in Table 1. To ensure good sampling adequacy for the factor analysis, the Kaiser-Olkin Measure of Sampling Adequacy and Bartlett’s Test of Sphericity were used (Dziuban & Shirkey, 1974). An exploratory factor analysis (EFA) was conducted with Promax rotation because factors were expected to correlate. Observations with missing values were excluded (*N* = 22). Extraction of factors was based on the theoretical dimensions, Kaiser-Guttman criterion (Kaiser, 1960) with eigenvalues > 1, in conjunction with a visual inspection of the Cattell’s scree plot (Cattell, 2012). A second estimation was then conducted using a pre-determined number of factors, based on the number of factors extracted in the first analysis, and a criterion for factor loadings of 0.40^1^. Cross-loaded items, or items loaded onto more than one factor, was removed from the model. The internal consistency of the subscales was determined using Cronbach’s α. Corrected item-total correlations were used as an indicator of homogeneity, with values from 0.30 to 0.70 considered acceptable (Ferketich, 1991). Lastly, correlations between the established AAQ factors and each of the HAB subscales were assessed with Pearson Correlation coefficients. All analyses were completed with IBM SPSS Statistics V. 28.

## Results

### Exploratory Factor Analyses

An EFA was conducted to determine the construct validity of the appraisal measure. The Kaiser-Meyer-Olkin Measure (KMO) of Sample Adequacy, was found to be higher than 0.50 at 0.87, indicating that the sample was sufficient for the 22-item scale. Higher KMO values indicate greater adequacy of the data for performing factor analysis (Shrestha, 2021). Bartlett’s Test of Sphericity gave a *p-*value of < 0.001, suggesting the data was suitable for factor analysis.

The results of the EFA are shown in Table 3. The total variance explained table was observed and was identified that six factors were greater than 1 eigenvalue and 3 factors were greater than 2 eigenvalues in the 22-item scale. A visual inspection of the scree plot, a second estimation using a pre-determined number of three factors, and factor loadings greater than 0.40 confirmed a 3-factor structure. Each factor was labelled to represent appraisal dimensions that the included items were making up. The first factor was composed of eight items related to Emotional Distress (e.g., Thinking about the event makes me feel guilty). Eight items related to Perceived Controllability (e.g., The event was expected. Meaning, I knew the event was going to happen) loaded onto the second factor, and the third factor was made up of the six items related to Perceived Threat (e.g., The event is important for me because I felt that my life was threatened because of the event). One item (Thinking about the event makes me feel pride) did not load onto any of the factors, and was thus dropped. All other primary factor loadings were ≥0.41, and there was one substantial cross-loading (Item 15; greater than 0.30) which was also dropped (Costello & Osborne, 2005). Item 2 (The event is important for me because it had a positive impact on my life) of the third scale is a reverse item.

**Table 3:** Exploratory factor analysis of the adversity appraisal questionnaire (*n* = 105)^*a*^

AAQ Item #	AAQ Item	Factor 1: Emotional Distress	Factor 2: Perceived Controllability	Factor 3: Perceived Threat
13	Thinking about the event makes me feel lonely	0.73		
14	Thinking about the event makes me feel angry	0.64		
16	Thinking about the event makes me feel embarrassed	0.91		
17	Thinking about the event makes me feel anxious/afraid	0.85		
18	Thinking about the event makes me feel sadness/grief	0.66		
19	Thinking about the event makes me feel guilty	0.95		
20	Thinking about the event makes me feel shameful	0.99		
22	Thinking about the event makes me feel confused	0.81		
4	The event is important for me because it had a lasting impact for me.		0.59	
6	The event is important for me because it happened to me because of the sort of person I am.		0.74	
8	The event is important for me because I was responsible for what happened.		0.64	
9	I understand what happened and what the consequences were.		0.51	
10	When it happened, I could predict how the event was going to end.		0.72	
11	The event was expected. Meaning, I knew the event was going to happen.		0.74	
12	Things I expected to happen during the event did actually happen.		0.72	
1	The event is important for me because it affected someone else’s well-being.			0.62
2	The event is important for me because it had a positive impact on my life despite it being a difficult experience.			− 0.41
3	The event is important for me because it had a negative impact on my life.			0.79
5	The event is important for me because I felt that my life was threatened because of the event.			0.59
7	The event is important for me because I couldn’t stop this event from happening to me.			0.82
15	Thinking about the event makes me feel happy/joyful		0.46	− 0.45
	Initial Eigenvalues	8.89	2.70	2.01
	% of Variance	40.41	12.28	9.14
	Cumulative %	40.41	52.68	61.82

^a^ Promax rotation (extraction eigenvalues > 1 and scree test)

### Reliability

Cronbach’s α coefficients were examined to test the reliability of the AAQ (Christmann & Van Aelst, 2006). The Cronbach’s α coefficients of each of the generated factors of the final three-factor model were obtained, which ranged from acceptable to high reliability of 0.94, 0.79, and 0.75 for the Emotional Distress, Perceived Controllability, and Perceived Threat factors respectively (Table 4). Significant correlations between subscales were found between subscales (Perceived Controllability-Emotional Distress: *r* = -.42, *p* <.001; Perceived Threat-Emotional Distress: *r* =.54, *p* <.001; Perceived Threat-Perceived Controllability: *r* = -.31, *p* <.01; see Table 5). The results of the corrected item-total correlation values of the scale items, are found to be moderate to high, ranging from 0.36 to 0.83 (Table 4).

**Table 4:** Reliability estimates for each subscale items of the adversity appraisal questionnaire

Factor	AAQ Item #	Corrected item-total correlation	Cronbach’s α
Emotional Distress	13	0.80	0.94
	14	0.82	
	16	0.76	
	17	0.68	
	18	0.83	
	19	0.77	
	20	0.82	
	22	0.83	
Perceived Controllability	4	0.44	0.79
	6	0.48	
	8	0.58	
	9	0.51	
	10	0.44	
	11	0.63	
	12	0.62	
Perceived Threat	1	0.46	0.75
	2	0.47	
	3	0.67	
	5	0.64	
	7	0.36	

**Table 5:** Correlations between AAQ factors and HAB

	1	2	3	4	5	6
(1) General Hostile Attributions	-	-	-	-	-	-
(2) General Hostile Responding	0.68***	-	-	-	-	-
(3) Child-Specific Hostile Attributions	0.68***	0.53***	-	-	-	-
(4) Factor 1: Emotional Distress	0.34**	0.26*	0.27*	-	-	-
(5) Factor 2: Perceived Controllability	0.05	0.04	0.07	− 0.42***	-	-
(6) Factor 3: Perceived Threat	− 0.06	0.04	− 0.06	0.54***	− 0.31**	-

*p* <.05, ** *p* <.01, *** *p* <.001

### HAB and Appraisal Factors

Bivariate correlations between each of the three appraisal factors (i.e., Emotional Distress, Perceived Controllability, and Perceived Threat) and HAB subscales (i.e., General Hostile Attributions, General Hostile Responding, and Child-Specific Hostile Attributions) are shown in Table 5. Emotional distress appraisals were significantly positively correlated with participants’ HAB scores, including general hostile attributions (*r =*.34, *p* <.01), general hostile responding (*r =*.26, *p* <.05) and child-specific hostile attributions (*r =*.27, *p* <.05). Perceived Controllability appraisals and Perceived Threat appraisals had null associations with all HAB subscales.

## Discussion

The present study sought to examine the factor structure of the Adversity Appraisal Questionnaire (AAQ), a newly developed measure that aims to document how individuals appraise adverse life events. The AAQ was designed to be broad in scope, aiming to capture multiple appraisal dimensions that may be relevant to a wide range of experiences. This novel tool sought to overcome limitations present in existing measures that only assessed appraisal of a single selected adverse experience, which was often a highly specific type of adversity. Cronbach’s α indicated adequate internal consistency and reliability, exceeding 0.70 for all factors, a value commonly accepted as a benchmark for sufficient reliability (Taber, 2018). The corrected-item total correlations indicated satisfactory item homogeneity. The construct validity was examined using an EFA, which indicated that the AAQ yields a reliable and clear three-factor model that represent the appraisal dimensions of Emotional Distress, Perceived Controllability, and Perceived Threat. Two items were removed due to low factor loading and cross-loadings. Ultimately, the final version of the AAQ included 20 items, each of which captures a distinct and relevant appraisal dimension. Indicating strong potential to reliably assess for core patterns of appraisal across multiple events, the resulting dimensions are consistent with the categories of appraisal conceptualized by appraisal theorists (Lazarus & Folkman, 1984).

The three factors uncovered for the AAQ are consistent to some extent with domains found in other appraisal measures that exist in the field. For example, the SAM also consists of threat dimensions as well as three dimensions of controllability (Controllable-by-self, Controllable-by-others, and Uncontrollable) that are also represented by the AAQ factors found in the present study. However, the domains of the AAQ are broader compared to that of other appraisal measures in existence. For example, the TAQ has separate scales for alienation, anger, and shame, which each contains several items. On the contrary, the AAQ has one item for each emotional response, which is loaded onto one broad scale (i.e., emotional distress). This would substantially reduce the length of the measurement tool and the subsequent fatigue that participants may feel.

Correlation analyses revealed that perceived Controllability and Emotional Distress were negatively correlated. This relation was also found in previous studies with adult samples who demonstrated high perceived controllability over breast cancer by asserting behavioral control (e.g., control by preventive behavior such as early detection through screening and detection) and showed less emotional distress (Decruyenaere et al., 2000). Another study found that higher perceived control mediated the relation between financial strain and emotional distress (Caplan & Schooler, 2007). These studies support the findings that Perceived Controllability and Emotional Distress are correlated, but separate concepts. Moreover, the perceived controllability of the adverse event could affect the degree to which individuals even attempt to cope with such situations (master vs. adjust). This suggests that individuals who perceive circumstances to not be controllable may experience emotional distress because they are not motivated to try to change their situation.

Findings also indicated that Perceived Threat and Emotional Distress were positively and significantly correlated, and that Perceived Threat and Perceived Controllability were negatively and significantly correlated. These relations are also supported by previous studies that found that adults who demonstrated higher perceived threat of the COVID-19 pandemic experienced greater emotional distress and unhealthy subjective mental well-being (Paredes et al., 2021). This correlation was again observed by another study which found that perceived threat of COVID-19 risks were exacerbated by overexposure to COVID-19 information, which in turn amplified emotional distress (Feng et al., 2022). Taken together, these studies support findings that Perceived Threat and Emotional Distress are correlated, and suggest that external environmental aspects may amplify an individuals’ perceived threat to a particular event. Empirical evidence also found the negative correlation between perceived threat and controllability in a sample of adults who showed that greater perceived threat of job insecurity was negatively correlated to perceived control (Vander Elst et al., 2014). Appraisal theory (Lazarus & Folkman, 1984) suggests that this relation exists because perceived threat increases when individuals feel that the pressures of daily and/or acute life stressors surpass their capacity to manage them.

Appraisal patterns across adverse events may also differ by one’s reported hostile attribution biases (HAB) (Dodge et al., 2015). Specifically, results showed that those who had stronger appraisals of emotional distress across adverse life events also reported greater tendencies to have HAB, for all types (i.e., general hostile attributions, general hostile responding, and child specific hostile attributions). However, other appraisal factors did not have significant associations with any of the HAB scales. Similar trends have also been observed by prior research that found emotional elements such as emotional distress and HAB may have a potential connection, and that the emotions and cognitive component that stem from adverse experiences may be processed through similar mechanisms (Dodge et al., 2015). To illustrate, research has indicated that HAB and emotional distress were both associated with physical and relational aggression such that aggressive individuals reported greater feelings of emotional distress and increased levels of HAB (Crick et al., 2002). Such findings are important because it gives a preliminary exploration of how appraisal styles of significant and traumatic events are also related to biased interpretations of cues in daily social activities and interactions. In this light, the study of appraisal may be an important point in which cognitive, emotional, and behavioral information intersect (Tuente et al., 2019), warranting further empirical attention.

## Future Directions and Conclusions

Despite the importance of understanding individuals’ subjective interpretations of adverse events, there does not appear to be a strong tool for assessing the complex and multidimensional nature of adults’ appraisal. The AAQ is unique in that it is a comprehensive scale that can be administered to populations with exposure to a broad range of events, which could be useful for understanding how appraisal patterns affect later adjustment. Moreover, the AAQ encompasses both primary and secondary categories of appraisal, a strength over a majority of other measures on adult appraisal that focus exclusively on primary appraisal (Gusler et al., 2021).

Despite the strengths of the study, it also has its limitations. The relatively small sample size made it possible for only an EFA and not a subsequent CFA to be conducted. Conducting a CFA on a different sample to validate the factorial structure derived from the results of this study is advised for future researchers. The approach was also cross-sectional, and therefore did not evaluate test-retest reliability or predictive validity. Moreover, the analytic sample was not nationally representative across demographic characteristics. Most participants likely lived in low-SES environments, identified as Black/African American or multiracial, and were recruited from a Midwestern urban setting. As such, the study findings may not be generalizable to all individuals who have adverse experiences, such as those living in different geographic areas. Despite its limitations, the findings of the current study suggest that the AAQ can be utilized to investigate appraisal styles related to adversity across multiple events. In addition, the level of exposure in the sample and range of events suggest that the measure captures a breadth not commonly found in other measurement tools that appraise adversity. This is a noteworthy contribution to the existing literature, as the field is in need of a tool that can capture relevant appraisals for various experiences and assess appraisal styles across events.

As is the case with any new scale, replication is recommended. In particular, due to the fairly selective nature of the sample (primarily Black low-income caregivers with high adversity exposure), the generalizability and utilization of the AAQ needs further replication with other samples from different population groups. Investigators might also compare the AAQ dimensions with other similar measures with similar constructs. For example, the relations between the AAQ and the Appraisal of Life Events scale (ALE; Ferguson et al., 1999) and the Appraisal of Illness Scale (Munkres et al., 1992) could provide some evidence of concurrent validity as subcomponents on the ALE and the Appraisal of Illness Scale have trauma appraisal scales, which might provide meaningful information about the Perceived Threat dimension of the AAQ. Finally, in using the scale, each subscale represents distinct domains of appraisals that are not necessarily on the same continuum. As such, using it as a total score would not be appropriate.

## Footnotes

1 This second estimation has been done on the same sample to confirm that the factor structure remained the same as the first estimation. This is not a CFA, as any reanalysis of the same sample essentially makes it exploratory.
